# Identification of Aging‐Related Hub Genes (ATP11B, RBBP7, DOCK10, and NUP160) as Potential Biomarkers and Therapeutic Targets in Sepsis

**DOI:** 10.1155/humu/9789556

**Published:** 2025-12-04

**Authors:** Xueyi Sun, Shaolei Geng, Zeyuan Wang, Qingjiang Chen

**Affiliations:** ^1^ Department of Rehabilitation Medicine, The First Affiliated Hospital of Zhengzhou University, Zhengzhou, China, zzu.edu.cn; ^2^ School of Basic Medical Sciences, Xinxiang Medical University, Xinxiang, Henan, China, xxmu.edu.cn; ^3^ Department of Oncology, The First Affiliated Hospital of Zhengzhou University, Zhengzhou, China, zzu.edu.cn

**Keywords:** aging-related genes, functional enrichment analysis, immune biomarkers, sepsis

## Abstract

Sepsis arises from a dysregulated host response to infection, leading to multiorgan inflammatory injury. Early diagnosis and treatment necessitate the identification of reliable immune biomarkers. This study investigated the relationship between aging, immunity, and sepsis by analyzing six human aging‐related gene sets (656 genes). We identified 16 aging‐related differentially expressed genes (DEGs) in sepsis. Among these, ATP11B, RBBP7, DOCK10, and NUP160 demonstrated the strongest connectivity with other genes and exhibited significant predictive power. Functional enrichment analysis (GO and KEGG) revealed distinct signaling pathway profiles between high‐risk and low‐risk sepsis groups (stratified based on risk scores). These dysregulated pathways, associated with multiple immune cells, were primarily linked to transcriptional dysregulation in cellular processes and cancer‐related pathways. Experimental validation assays corroborated the roles of ATP11B and RBBP7. Collectively, our bioinformatic and experimental findings indicate that ATP11B, RBBP7, DOCK10, and NUP160 are implicated in the pathogenesis and progression of sepsis. But their potential for sepsis biomarkers still requires further verification.

## 1. Introduction

Sepsis results from physiological, pathological, and metabolic problems induced by infection and has emerged as a global health issue [1]. The global burden is challenging to ascertain accurately; over 48.9 million individuals were diagnosed with sepsis, resulting in 11 million fatalities (20% of worldwide deaths) from sepsis in 2017 [2]. Although several factors increase sepsis mortality risk, aging plays a significant role [3, 4]. Older individuals are at a heightened risk of acquiring sepsis, with advancing age being a significant risk factor for sepsis‐related morbidity and mortality [5, 6]. High mortality rates result from poor prognosis due to late diagnosis. Therefore, approaches for early diagnosis are vital. Currently, only symptomatic treatment is applied. Immune system dysregulation in sepsis cannot be ignored. The host immune system reacts to infection through an early inflammatory response [7, 8]. Immunotherapy has emerged as a prevalent approach for oncological treatment [9, 10]. Recognizing that sepsis‐related immune cell deficiencies are associated with long‐term mortality risks, current research focuses on the potential of immunotherapy to improve long‐term outcomes in patients with sepsis [7, 8].

Previous work reported sepsis biomarkers by focusing on the transcriptome and clinical indicators [11–13]. A study indicated a correlation between septic shock and acute kidney injury (AKI), revealing that the genes PTX3, VMP1, OLFM4, SLPI, TIMP1, LCN2, and S100A9 are strongly correlated with novel biomarkers implicated in the onset and progression of sepsis‐associated acute kidney injury (SSAKI) [14]. A separate investigation demonstrated a significant correlation between the functional activity of MAP 2K2 and IRF7 in sepsis‐induced ARDS [15] [15]. Gong et al. identified LCK, TBX21, LRG1, ELANE, TP53, ZAP70, CD247, ITK, and FYN as promising novel biomarkers for early sepsis [16].

This study involved extensive bioinformatic analysis and animal trials, leading to the identification of novel gene biomarkers related to aging and immunity for immunotherapy and prognostic prediction in sepsis. These biomarkers offer novel opportunities for personalized diagnosis and therapy of sepsis.

## 2. Materials and Methods

### 2.1. Sepsis Dataset and Aging‐Associated Gene Set Collection

The sepsis dataset GSE65682 was acquired from the Gene Expression Omnibus (GEO) database. The collection comprised 802 blood samples from multiple cohorts, consisting of 760 sepsis specimens and 42 healthy controls. To mitigate potential batch effects arising from the multicenter and multiplatform nature of this dataset, we applied the “ComBat” algorithm from the “sva” R package for batch effect correction prior to downstream analysis. The subsequent bioinformatic analysis was conducted on the batch‐corrected dataset GSE65682 after sample standardization, annotation, and approval. We collected aging‐associated gene sets (the results were obtained by multifactorial ANOVA calculations for different species and tissues stratified by age) from [17]. The aging‐mediated disease alignment (AMDA) score was used to quantify the correlation between aging and sepsis. If aging aligns the transcriptome of a given disease, AMDA is considered positive; if aging diverges from a disease signature, AMDA is negative [17].

### 2.2. Calculation of Aging‐Related Indices in Patients With Sepsis

The set of aging‐related genes in humans was selected, and the intersection was analyzed. Based on the expression of the intersecting genes, gene set variation analysis (GSVA) [18] was performed to calculate the enrichment score, and the enrichment score was used as the aging‐related index (ARI) for patients with sepsis. Based on the Wilcoxon test, ARI was compared between patients with sepsis over and under 50 years of age.

### 2.3. Examination of Immune Cell Infiltration in Individuals With Sepsis

CIBERSORT was employed to quantify the amounts of 22 immune cell types in sepsis patients [19]. Pearson′s correlation analysis was conducted to ascertain the relationship between ARI and the amounts of different immune cells.

### 2.4. Analysis of Pathway Enrichment for Genes Associated With Aging

Enrichment analysis of aging‐related genes was conducted using Gene Ontology (GO) and the Kyoto Encyclopedia of Genes and Genomes (KEGG) to identify associated pathways.

### 2.5. ARI‐Related Drug Analysis

The samples were categorized into high and low groups based on the median ARI, and the limma program was employed to identify differential genes between the two groups. Small‐molecule inhibitors or inducers in the high ARI group were obtained from the CMap (https://clue.io/) database according to the upregulated and downregulated genes, and the mechanism of action (MOA) of the compounds was explored.

### 2.6. Identifying Genes With Differences in Expression in Patients With Sepsis Compared to Healthy Individuals

The limma software was employed to identify differentially expressed genes (DEGs) in patients with sepsis and healthy controls [20]. Genes that satisfied the criteria of |LogFC| > 0.5 and FDR < 0.05 were identified as DEGs. Pathway enrichment analysis of the DEGs was performed utilizing GO and KEGG.

### 2.7. Machine Learning Screens for Key Genes Affecting ARI

Based on the GSE65682 dataset, the random forest (RF) [21] model and least absolute shrinkage and selection operator (LASSO) [22] were used. ARI groups were considered as the response variables and DEGs as explanatory variables. Key genes influencing ARI were found through commonalities in RF and LASSO data, and their predictive capacity for ARI was verified via receiver operating characteristic (ROC) analysis. We first evaluated the importance score of the variables (based on MeanDecreaseGini) and then retained those with a score greater than 2 (to ensure biological relevance). For LASSO, we selected variables with nonzero coefficients using 10‐fold cross‐validation (min lambda = 0.04). The 16 common factors were the intersection of these two filtered sets. The Wilcoxon test was employed to assess the differential expression of important genes between the healthy and sepsis cohorts, with results illustrated through a heat map and box line plots.

### 2.8. Correlation of Key Genes and Immune Cells

The relationship between essential genes and 22 immune cells was examined, and the interrelations among the critical genes were investigated.

### 2.9. Finding Target miRNAs for Key Genes

The online mirTarbase (https://mirtarbase.cuhk.edu.cn/) database was used to identify target miRNAs corresponding to key genes, and the network was mapped using Cytoscape.

### 2.10. Building of Sepsis Model

MDL Biotech (Beijing, China) provided 12 adult male C57BL/6 J mice, weighing 30–40 g and aged 8–10 weeks. Mouse tests were performed in standard cages under controlled temperature and humidity conditions (22^°^C ± 2^°^C, 40%–60%, *n* = 5 per cage) during a 12‐h light/dark cycle with unrestricted access to food and water. Following a week of acclimatization, the mice underwent testing. The rats were randomly allocated into two groups (*n* = 6): the LPS group received a 10 mg/kg intraperitoneal injection of lipopolysaccharide (LPS), whereas the control group was administered normal saline. This study received approval from the Ethical Review Board of Zhengzhou University and adhered to the NIH Guidelines for Laboratory Animal Care and Use.

Mouse euthanasia was carried out using the cervical dislocation method. At 24 h postoperation, the mice were humanely sacrificed via cervical dislocation. Subsequently, blood samples were collected and subjected to analysis by RT‐PCR. The detailed procedure of euthanasia is described as follows: The mouse was first positioned on a foam board. The experimenter then grasped the mouse′s tail with the right hand and firmly immobilized the mouse′s head using the left thumb and index finger. In a controlled and rapid motion, the right hand pulled the tail in a posterior direction. This mechanical force induced cervical spine dislocation, leading to the humane and efficient termination of the mouse′s life, which complies with ethical and scientific standards for animal experimentation.

### 2.11. RT‐PCR Analysis

As previously documented, RT‐PCR analysis was conducted [23]. Total RNA was isolated from blood using TRIzol reagent. The RNA concentration and purity were assessed using UV spectroscopy. Subsequently, the TaqMan Reverse Transcription Kit and Gene Amp PCR were employed to synthesize cDNA. PCR was conducted using qPCR SYBR Green Master Mix. Each amplification was conducted thrice. Forward primers for ATP11B, RBBP7, DOCK10, and NUP160 are as follows: TGTGGAACAACATATAGACCCA, 5 ^′^‐TTTAATGCTCAAGAGACCGT‐3 ^′^, 5 ^′^‐TAGTTTCAGATTACGCAGGTC, and TCCTGCCTATCATCTTGAACC, respectively.

## 3. Results

### 3.1. AMDA Score for Sepsis

**Figure 1:**
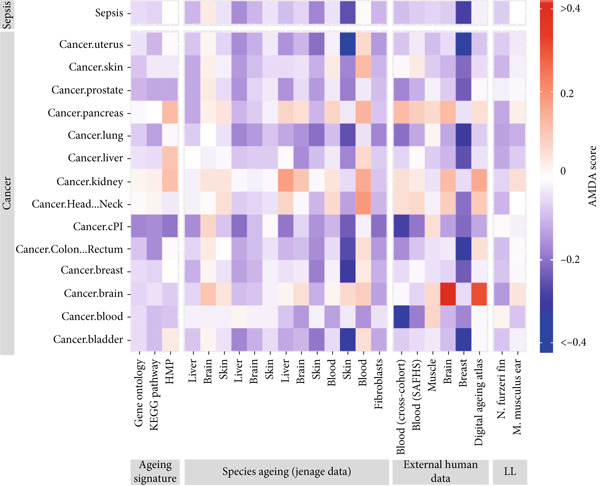
Comparison of AMDA scores between sepsis and different cancers.

The AMDA score for sepsis was largely negative (Figure 1). This suggested that aging was related to changes in the transcriptome and that such changes were reversed in the sepsis group versus the healthy control group. This feature of sepsis is similar to that of tumors (Figure 1), and Mirouse et al. also showed that both sepsis and tumors are caused by the inability of the host immune system to cope with the initial damage [24]. We believed that the aging microenvironment of sepsis and tumors has many commonalities.

### 3.2. ARI as an Index for Evaluating Aging

We identified 656 shared genes from six genomes linked with human aging and computed the ARI using GSVA based on the expression of these genes (Figure 2a). The ARI was markedly reduced in individuals with sepsis aged over 50 compared to those under 50 (Figure 2b), corroborating our prior conclusion.

Figure 2Aging‐related genes and ARI calculation. (a) Intersection of six human aging‐associated gene sets. (b) Differences in ARI in different age groups.

**Figure:**
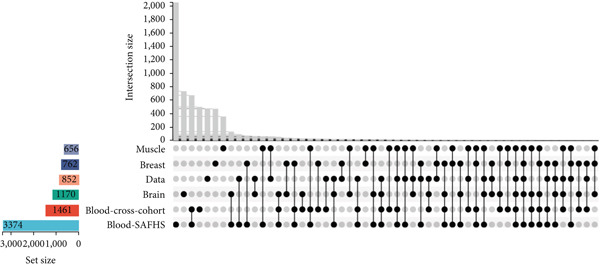
(a)

**Figure:**
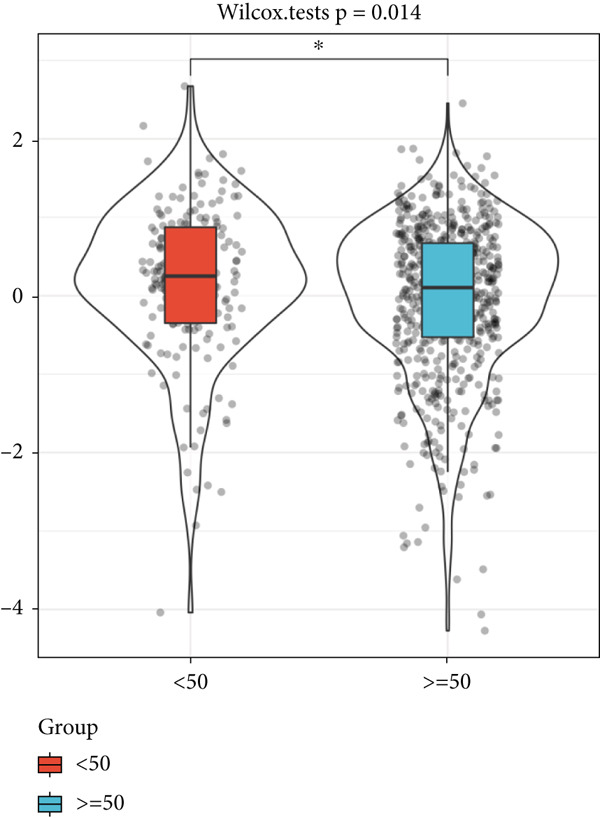
(b)

### 3.3. Association of ARI With Immune Cells

**Figure 3:**
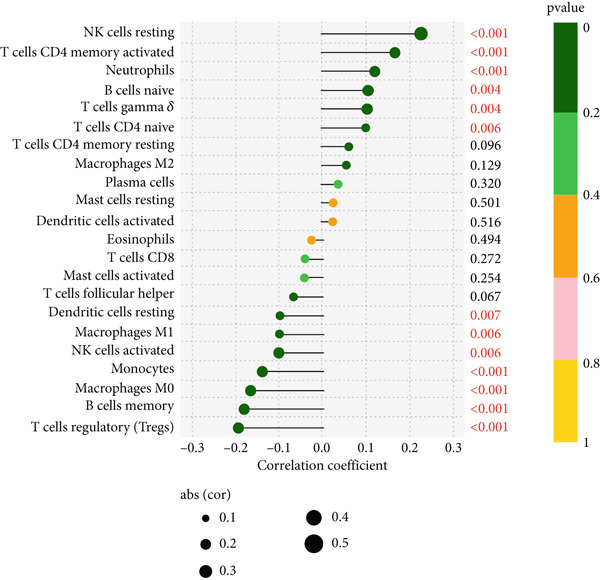
Correlation of ARI with immune cells.

Of the 22 immune cells analyzed by CIBERSORT, resting NK cells and activated memory CD4 T cells had a positive correlation with ARI, while regulatory T cells (Tregs) and memory B cells demonstrated a negative correlation with ARI (Figure 3).

### 3.4. Pathways Involved in Aging‐Related Genes

Our GO enrichment analysis revealed that aging‐related genes were predominantly associated with chromosomal organization and the regulation of cellular metabolic activities (Figure 4a). KEGG analysis indicated that genes associated with aging predominantly fell under cell cycle and tumor‐related pathways (Figure 4b).

Figure 4Pathway enrichment analysis of genes associated with aging. (a) Gene Ontology (GO) pathway enrichment analysis. (b) KEGG pathway enrichment study.

**Figure:**
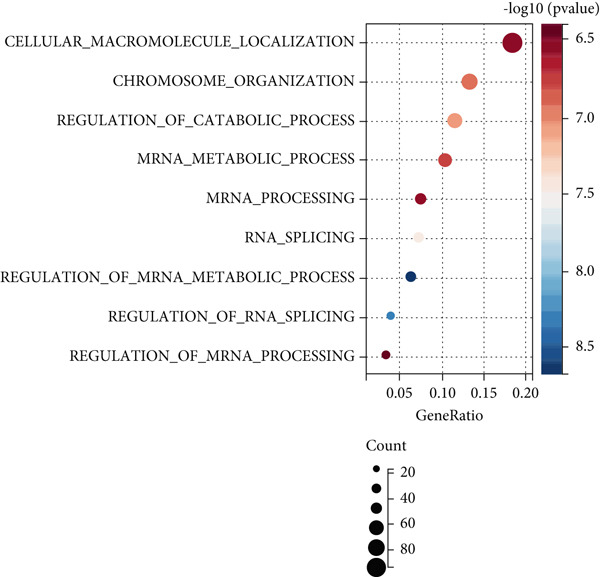
(a)

**Figure:**
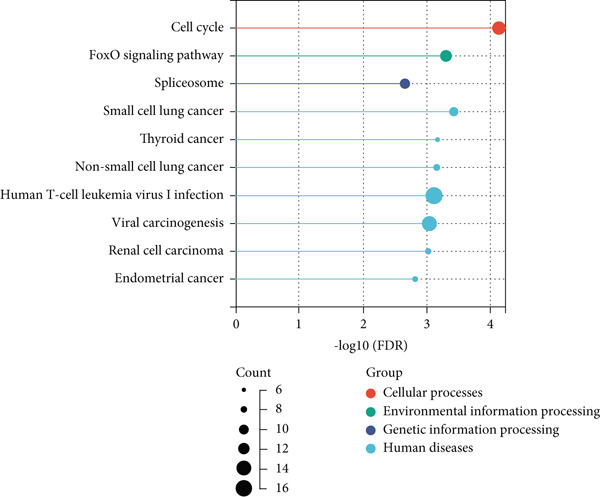
(b)

### 3.5. Compounds Affecting ARI

We screened 27 compounds sensitive to the high ARI group, and these compounds shared 22 molecular pathways (MOAs) (Table 1). Among them, three compounds had the same MOA as dopamine receptor agonist; six compounds had the same MOA as adrenergic receptor antagonist, cyclooxygenase inhibitor, and glucocorticoid receptor agonist (Figure 5a). We selected two compounds, isoflupredone (a glucocorticoid receptor agonist) and sulmazole (a phosphodiesterase inhibitor and calcium sensitizer), for structural depiction (Figure 5b,c). These two were chosen not only because they were among the top‐ranked compounds by enrichment score but also because they represent distinct and therapeutically relevant MOAs among the highly enriched compounds, highlighting the diversity of potential pharmacological interventions targeting the high‐ARI state.

**Table 1:** Compounds sensitive to the high ARI group.

**Name**	**Description**	**MOA**	**Enrichment**
Ambroxol	Sodium channel blocker	Sodium channel blocker	0.66
Amitriptyline	Norepinephrine inhibitor	Norepinephrine inhibitor, norepinephrine reuptake inhibitor, serotonin receptor antagonist, serotonin reuptake inhibitor	0.61
Anisomycin	DNA synthesis inhibitor	DNA synthesis inhibitor	0.769
Cinchonine	P‐glycoprotein inhibitor	P‐glycoprotein inhibitor	0.808
Doxylamine	Histamine receptor antagonist	Histamine receptor antagonist	0.7
Fludrocortisone	Glucocorticoid receptor agonist	Glucocorticoid receptor agonist, mineralocorticoid receptor agonist	0.505
Genistein	Tyrosine kinase inhibitor	Tyrosine kinase inhibitor	0.426
Isoflupredone	Glucocorticoid receptor agonist	Glucocorticoid receptor agonist	0.901
Isoxicam	Cyclooxygenase inhibitor	Cyclooxygenase inhibitor	0.685
Karakoline	Phytotoxin	Phytotoxin	0.52
Ketotifen	Histamine receptor agonist	Histamine receptor agonist, histamine receptor ligand, leukotriene receptor antagonist, phosphodiesterase inhibitor	0.701
Lasalocid	Bacterial permeability inducer	Bacterial permeability inducer	0.767
Lisuride	Dopamine receptor agonist	Dopamine receptor agonist	0.594
Memantine	Glutamate receptor antagonist	Glutamate receptor antagonist	0.65
Metronidazole	DNA inhibitor	DNA inhibitor, antiprotozoal	0.567
Nadolol	Adrenergic receptor antagonist	Adrenergic receptor antagonist	0.82
Naltrexone	Opioid receptor antagonist	Opioid receptor antagonist	0.619
Naringenin	Aromatase inhibitor	Aromatase inhibitor, TRPV antagonist	0.68
Phenazone	Cyclooxygenase inhibitor	Cyclooxygenase inhibitor	0.876
Podophyllotoxin	Microtubule inhibitor	Microtubule inhibitor, tubulin inhibitor	0.668
Puromycin	Protein synthesis inhibitor	Protein synthesis inhibitor	0.626
Quinpirole	Dopamine receptor agonist	Dopamine receptor agonist	0.707
Quinpirole	Dopamine receptor agonist	Dopamine receptor agonist	0.707
Rifampicin	RNA polymerase inhibitor	RNA polymerase inhibitor	0.726
Sulmazole	Adenosine receptor antagonist	Adenosine receptor antagonist	0.907
Thapsigargin	ATPase inhibitor	ATPase inhibitor	0.774
Timolol	Adrenergic receptor antagonist	Adrenergic receptor antagonist	0.672

Figure 5Compounds sensitive to the high ARI group. (a) Compounds sensitive to the high ARI group and their MOA. (b) Molecular structure of isoflupredone. (c) Molecular structure of sulmazole.

**Figure:**
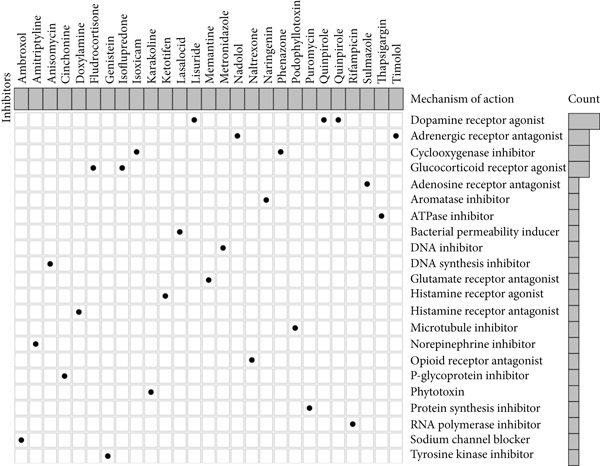
(a)

**Figure:**
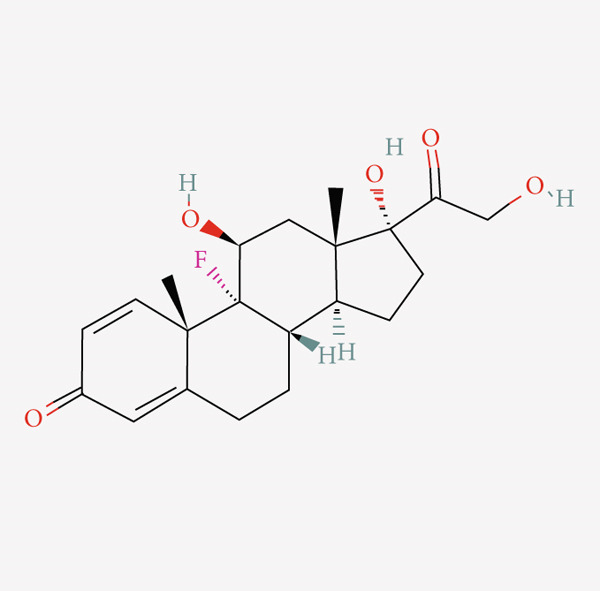
(b)

**Figure:**
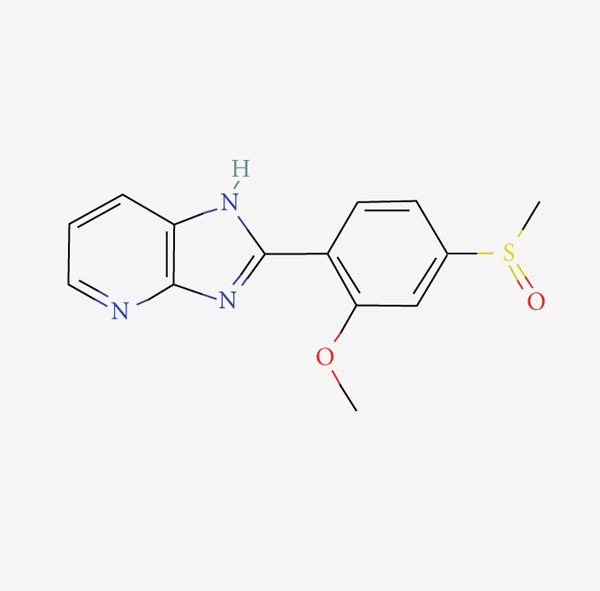
(c)

### 3.6. Dysregulated Genes in Sepsis

We obtained 317 dysregulated genes in patients with sepsis, of which 117 were upregulated in sepsis and 200 were downregulated (Figure 6a). Enrichment analyses performed with KEGG and GO indicated that the DEGs were predominantly associated with immune‐related pathways, including T cell activity, Th1 and Th2 cell differentiation, Th17 cell differentiation, and T cell receptor signaling pathways (Figure 6b,c).

Figure 6Differential genes in sepsis. (a) Volcano map of differential genes. (b) KEGG pathway enrichment analysis of differential genes. (c) GO pathway enrichment analysis of differential genes.

**Figure:**
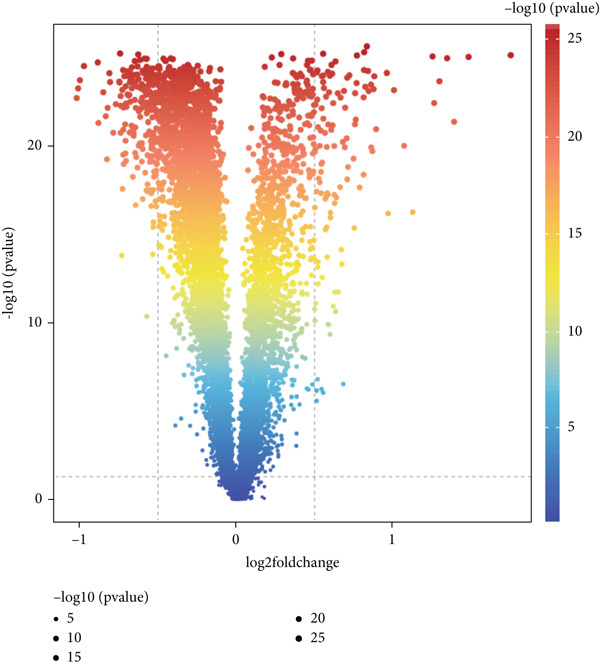
(a)

**Figure:**
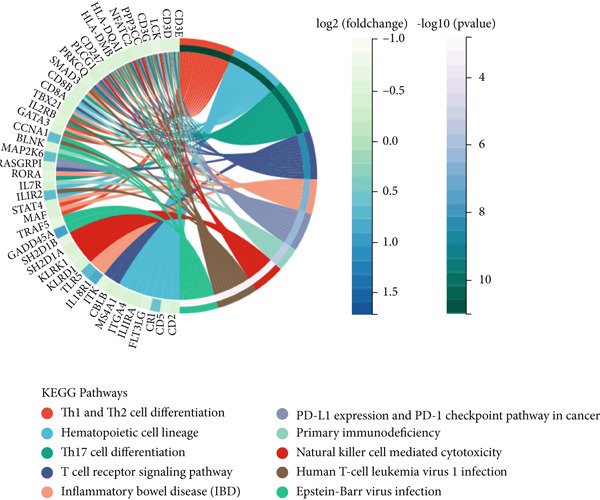
(b)

**Figure:**
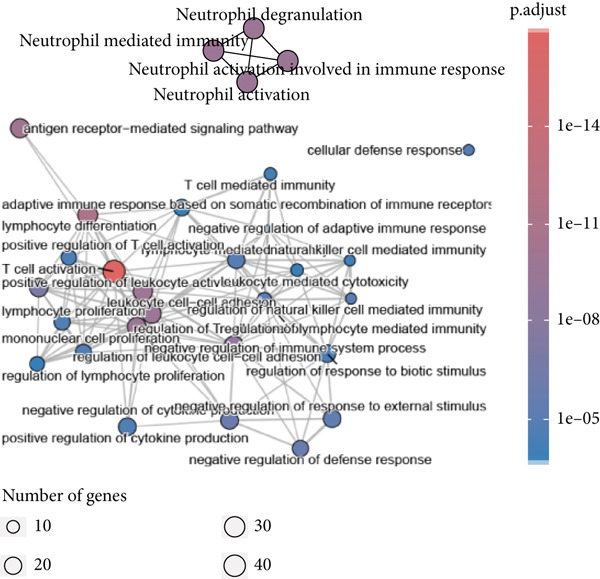
(c)

### 3.7. Important Identifiers for High and Low ARI Groups

Sixteen factors that could distinguish between high and low ARI groups were obtained by LASSO and RF (Figures 7a, 7b, and 7c), all of which had AUC values higher than 0.6. In addition, *ATP11B*, *RBBP7*, *DOCK10*, and *NUP160* had AUC values higher than 0.7 (Figure 7d). Among them, MEX3C had the highest AUC value of 0.862.

Figure 7Machine learning to obtain factors affecting ARI. (a, b) LASSO regression analysis and RF. (c) Common factors identified by LASSO and RF. (d) ROC curve for each significant factor.

**Figure:**
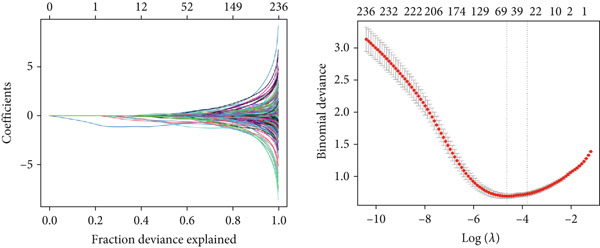
(a)

**Figure:**
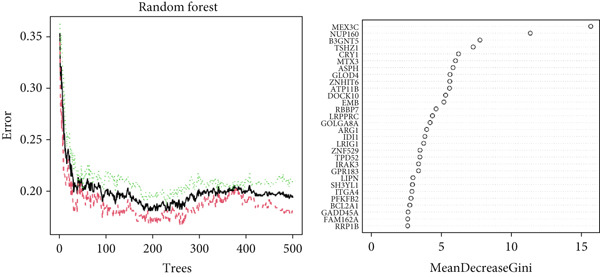
(b)

**Figure:**
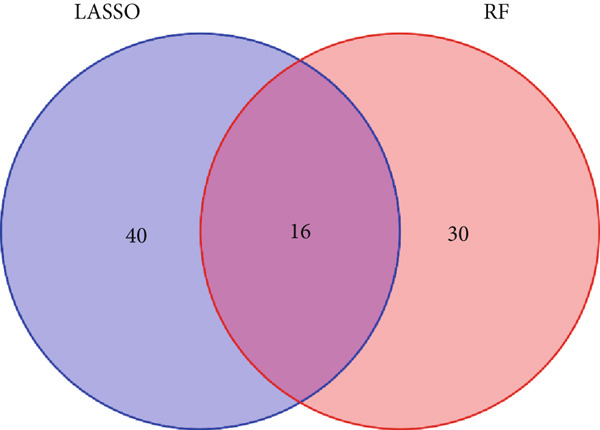
(c)

**Figure:**
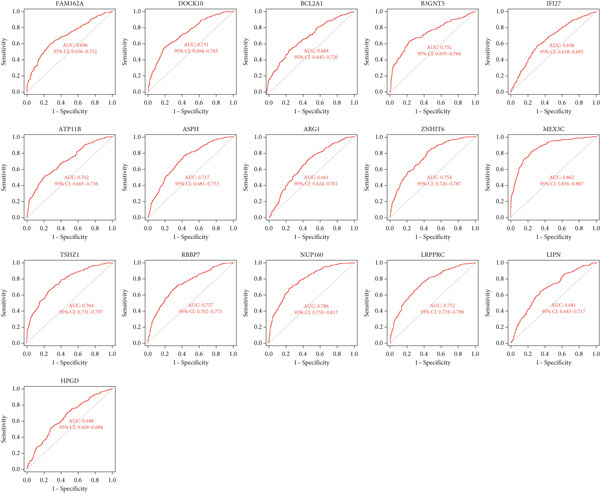
(d)

### 3.8. Differential Expression of Key Genes

The heat map shows that *FAM162A*, *RBBP7*, *MEX3C*, *TSHZ1*, *NUP160*, *ZNHIT6*, *DOCK10*, and *LRPPRC* were highly expressed in the healthy group, while *IFI27*, *ARG1*, *ASPH*, *HPGD*, *LIPN*, *BCL2A1*, *ATP11B*, and *B3GNT5* were highly expressed in the sepsis group (Figure 8a), and the differences were significant (Figure 8b).

Figure 8Differential expression of key genes. (a) Heat map of differential expression. (b) Box line plot of differential expression.  ^∗^ *p* < 0.05,  ^∗∗^ *p* < 0.01, and  ^∗∗∗^ *p* < 0.001.

**Figure:**
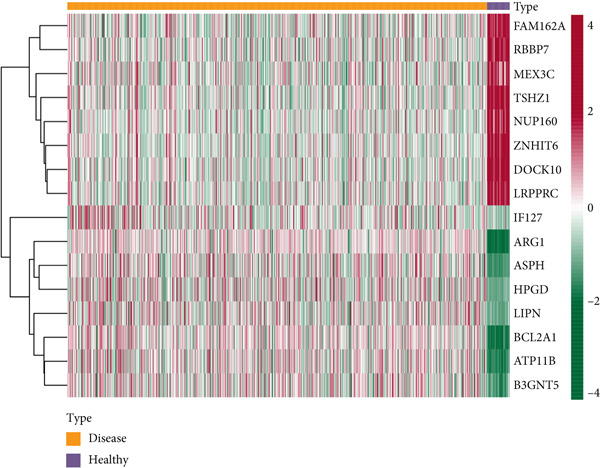
(a)

**Figure:**
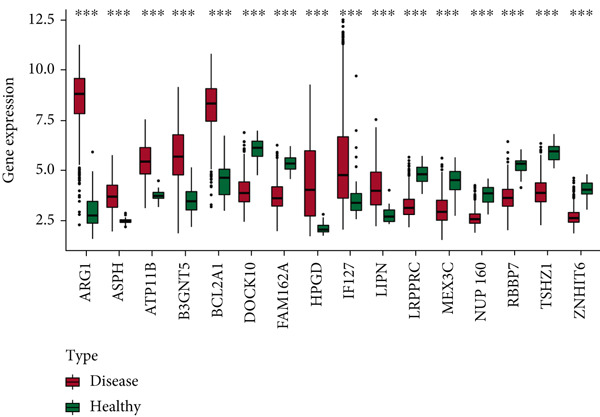
(b)

### 3.9. Association of Important Factors and Immune Cells

**Figure 9:**
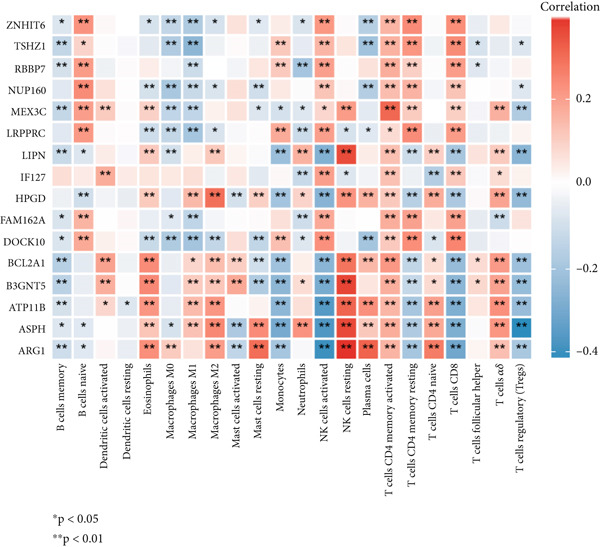
Correlation among 16 factors and 22 immune cells.

All 16 factors were positively correlated with T cells CD4 memory activated, while almost all these genes were negatively correlated with B cells memory. *MEX3C*, *LIPN*, *HPGD*, *BCL2A1*, *B3GNT5*, *ARG1*, *ASPH*, and *ATP11B* had a strong positive correlation with NK cells resting (Figure 9).

### 3.10. Interconnection Among the 16 Factors

We also carried out interconnection analysis and found that these 16 factors were all positively correlated with each other, among which *ATP11B*, *RBBP7*, *DOCK10*, and *NUP160* were more closely associated with other genes (Figure 10a).

Figure 10Correlation of key genes and differences in expression. (a) Interconnection among the 16 factors. (b) mRNA–miRNA regulatory network. (c) mRNA expression levels of genes in the C and LPS groups. Each point represents the mean ± SD of three independent replicates;  ^∗^ *p* < 0.05 (Student′s *t*‐test).

**Figure:**
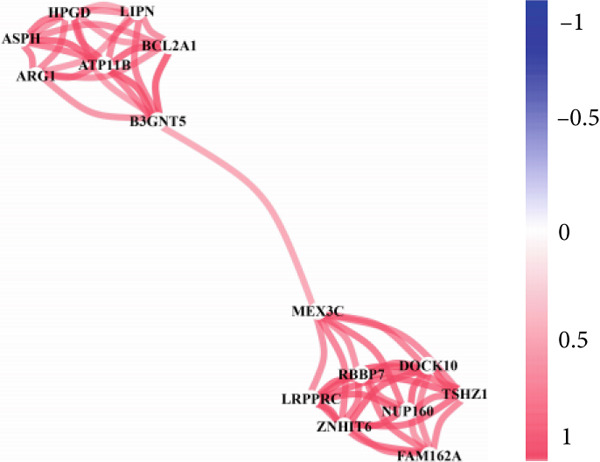
(a)

**Figure:**
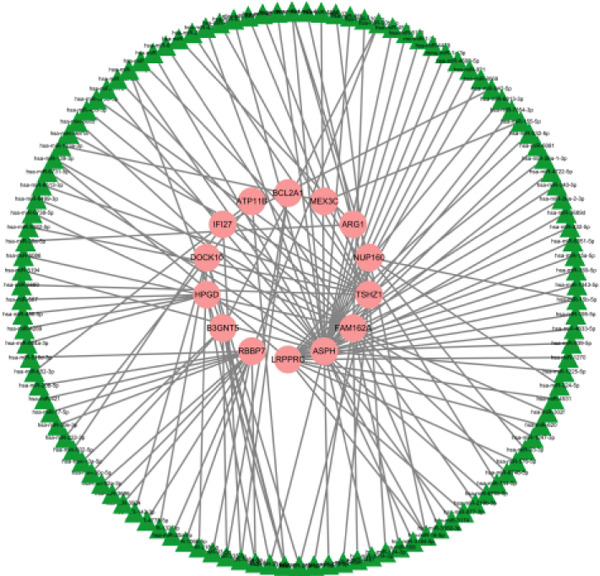
(b)

**Figure:**
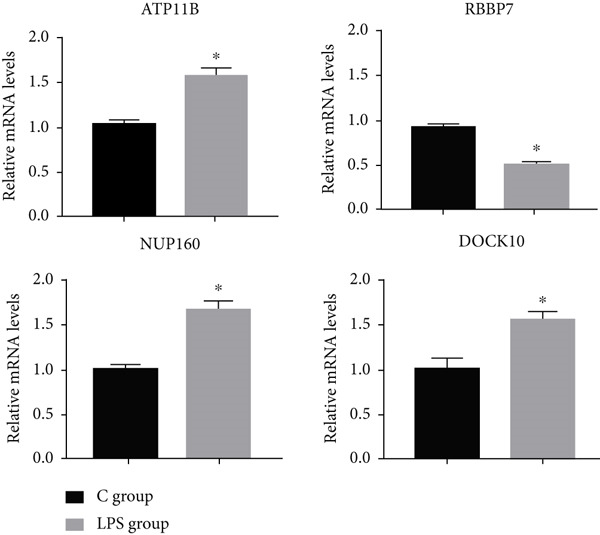
(c)

### 3.11. mRNA–miRNA Regulatory Network

We mapped the mRNA–miRNA network using the mirTarbase database. To quantitatively identify hub genes within this network, we calculated the degree centrality (number of connecting miRNAs) for each mRNA. The genes ASPH (degree = 12), LRPPRC (degree = 10), TSHZ1 (degree = 9), FAM162A (degree = 8), and RBBP7 (degree = 7) were identified as the top five hubs with the highest connectivity, substantiating our claim that they have “more target miRNAs” (Figure 10b).

### 3.12. RT‐PCR Analysis to Verify the Expression of Key Genes

This work demonstrated that mRNA expressions of ATP11B were significantly elevated and those of RBBP7 were significantly decreased in LPS‐induced sepsis, aligning with the findings of bioinformatic analysis. The mRNA expression of NUP160 and DOCK10 was elevated in LPS‐induced sepsis, although the bioinformatic analysis indicated a contrary trend (Figure 10c). The reasons for the differences between the two may include the following points. (1) Species differences: The bioinformatic analysis used human sepsis datasets, while validation was performed in mice; conserved versus species‐specific regulation of these genes may differ [25]. (2) Pathological stage divergence: The LPS model reflects acute sepsis, whereas human datasets may include heterogeneous stages (e.g., early vs. late sepsis), potentially altering expression trends [26]. (3) Posttranscriptional regulation: miRNAs or RNA‐binding proteins may suppress translation in the healthy group despite high transcript levels, leading to discordant protein versus mRNA trends [27]. We emphasize that NUP160/DOCK10 require further validation in human clinical samples and functional studies (e.g., luciferase assays for miRNA binding) before being considered robust biomarkers. Each point denotes the mean ± standard deviation of three independent replicates;  ^∗^ *p* < 0.05 (Student′s *t*‐test).

## 4. Discussion

The AMDA score for sepsis was largely negative (Figure 1), suggesting that the transcriptomic changes associated with aging are reversed in sepsis compared to healthy controls. This feature is superficially similar to that observed in many tumors (Figure 1). Mirouse et al. also highlighted that both sepsis and tumors involve a failure of the host immune system to adequately respond to initial damage [24]. However, it is crucial to acknowledge the fundamental mechanistic distinctions behind this shared “reversal” phenotype. In cancer, the negative AMDA score often reflects proliferative and antisenescence pathways that drive tumorigenesis. In contrast, in sepsis, it more likely signifies a global disruption of homeostasis, massive immune cell death, and a shift toward a dysfunctional or immunosuppressive state, rather than a pro‐proliferative program. Thus, while the transcriptomic “reversal” of aging signatures is a common endpoint, the underlying pathophysiological drivers in sepsis and cancer are distinct.

The importance of aging and immunity in tumorigenesis is established [28–30]; however, their roles in sepsis are still unclear. Sepsis is one of the main reasons for admission to the intensive care unit in older adults [31] and could cause multiple immune deficiencies, leading to prolonged inflammation, immunosuppression, susceptibility to infection, and insurmountable death [7]. Inhibiting inflammation entails a variety of interconnected, intricate, and coordinated cellular processes and previously identified chemical signals. Damaged tissues, cells, and leukocytes must be eliminated from the infection site promptly after the pathogen is eradicated from the host. In optimal conditions, defunctionalized histiocytes and leukocytes suffer apoptosis and are phagocytosed by macrophages, leading to their removal from the inflamed region and the subsequent production of anti‐inflammatory interleukin‐10 (IL‐10) and transforming growth factor beta (TGF‐*β*). Moreover, recently identified bioactive lipids, namely, lipoxins, resolvins, protectins, and maresins, have demonstrated the capacity to diminish reactive oxygen species (ROS), endothelial permeability, and leukocyte recruitment, while enhancing macrophage phagocytosis [32, 33]. Alongside anti‐inflammatory cytokines, the resolution of inflammation is regulated by various subsets of immune cells, including Tregs [34, 35] and myeloid‐derived suppressor cells, which orchestrate the inhibition of cytotoxic effectors and the suppression of inflammatory cytokine production [36]. Despite reduced myeloid synthesis of proinflammatory cytokines, total myeloid production and release of anti‐inflammatory cytokines such as IL‐10 are increased [37]. The apoptosis of lymphocytes and antigen‐presenting cells (dendritic cells and B cells) is regarded as a characteristic of immunological suppression in sepsis [38, 39]. Natural killer (NK) cells demonstrate their immunological role within a regulatory immune complex. NK cells are categorized into distinct subpopulations according to the expression of CD16 and CD56 on their surface [40]. Both CD56^hi^ and CD56^low^ NK cell subsets are significantly altered during sepsis. NK cell activation protects against sepsis‐associated pneumonia by inhibiting bacterial growth and overproduction of INF‐*γ* which has important implications for sepsis prognosis [41]. Therefore, we explored novel methods for diagnosis and treatment related to aging and immunity in sepsis.

We obtained an aging‐related gene set calculated by age‐stratified multivariate ANOVA of different species and tissues using the GSE65682 dataset from the GEO database. The AMDA score in sepsis was calculated to quantify the relationship between aging and disease and compared with the AMDA score of tumors. Similarities and differences were also confirmed. AMDA scores were positive if changes in senescence and aging‐related transcriptomes in the disease group were consistent with changes in those genes in healthy controls, and otherwise, AMDA scores were negative. Gene sets associated with aging in humans were identified, and their intersection was utilized to find significant aging‐related genes. The enrichment scores for each patient with sepsis‐associated pneumonia were derived from gene expression using GSVA, serving as the ARI. Subsequently, we conducted GO and KEGG enrichment analyses on these aging‐related genes to identify associated pathways. We employed CIBERSORT to quantify the immune cell composition in sepsis patients and assessed the link between ARI and immune cells, utilizing the limma package to identify differential genes between the high and low ARI cohorts. Sixteen factors exhibited a positive correlation with other genes, with ATP11B, RBBP7, DOCK10, and NUP160 demonstrating a closer association with additional genes. The mRNA expression of ATP11B was increased, but RBBP7 was downregulated in LPS‐induced sepsis, aligning with the findings from the bioinformatic study. Nonetheless, the mRNA expression of NUP160 and DOCK10 was elevated in LPS‐induced sepsis, which contradicts the findings from the bioinformatic analysis. Despite the discordance in expression trends for NUP160 and DOCK10 between bioinformatic predictions and our murine validation, their potential as sepsis biomarkers cannot be dismissed. This discrepancy may stem from interspecies variation, differences in disease stage (acute LPS model vs. heterogeneous human sepsis), or posttranscriptional regulation. Therefore, further validation in human clinical cohorts and functional studies is essential to definitively ascertain their biomarker utility.

Another bioinformatic study showed that ATP11B expression might be associated with AKI and septic shock, with a change trend similar to ours [14]. And Wang et al. reported that ATP11B modified synaptic ultrastructure and promoted spine remodeling [42]. Low ATP11B expression was also associated with a worse prognosis and increased metastasis in breast cancer patients, according to Xu et al. [43]. Regulation of NUP160 expression could provide therapeutic benefit in some diseases such as diabetic nephropathy, angiosarcoma, and steroid‐resistant nephrotic syndrome [44–46]. One class of proteins that activate the Rho GTPase is the dock proteins. Three members of the Zizimin subfamily are Dock9, Dock10, and Dock11. Chronic lymphocytic leukemia (CLL) is characterized by the activation of the DOCK10 gene by interleukin‐4 (IL4) [47, 48]. However, we did not find any reports linking NUP160 and DOCK10 with sepsis. One bioinformatic analysis showed that RBBP7 can be used as a biomarker for sepsis, and the reported change trend was the same as ours [15]. According to Dave et al., it may provide a novel therapeutic target for tauopathies associated with Alzheimer′s disease [49]. Based on our bioinformatic and experimental findings, we further explored the therapeutic potential of ATP11B and RBBP7 within sepsis pathophysiology. Their correlations with specific immune cells (Figures 3 and 9) and involvement in dysregulated pathways (Figures 4 and 6) offer insights into how modulating their expression might influence sepsis progression. ATP11B, upregulated in sepsis, shows a negative correlation with memory B cells and a positive correlation with resting NK cells and activated memory CD4+ T cells (Figures 3 and 9), suggesting its role in regulating the transition from hyperinflammation to immunosuppression. Inhibiting ATP11B could potentially mitigate early hyperinflammation by modulating CD4+ T cell activation, while alleviating late‐stage immunosuppression by reversing its suppression of memory B cells—consistent with the enrichment of T cell receptor signaling pathways (Figure 6b,c). In contrast, RBBP7 is downregulated in sepsis and demonstrates positive correlations with activated NK cells and memory CD4+ T cells. As a chromatin remodeler, its downregulation may promote sepsis‐induced immunosuppression through epigenetic mechanisms. Restoring RBBP7 function could potentially enhance NK cell cytotoxicity and T cell‐mediated immunity against lymphocyte exhaustion, aligning with the transcriptional dysregulation pathways identified in KEGG analysis (Figure 4b).

Furthermore, the interplay between ATP11B and RBBP7 appears to form a fine‐tuning mechanism for immune balance. Their opposing effects on certain cell populations (e.g., memory B cells) suggest that targeting this regulatory axis might enable more precise restoration of immune homeostasis in sepsis, moving beyond broad immunosuppressive or immunostimulatory approaches.

## 5. Conclusions

*ATP11B*, *RBBP7*, *DOCK10*, and *NUP160* may play the major role in the occurrence and progression of sepsis. The mRNA expression of *ATP11B* was upregulated and that of *RBBP7* was downregulated in LPS‐induced sepsis, in line with our bioinformatic analysis results. However, the mRNA expression of *NUP160* and *DOCK10* was upregulated in LPS‐induced sepsis, which was contrary to the bioinformatic analysis results. Our research shows that ATP11B and RBBP7 emerge as validated, promising biomarkers for sepsis, supported by consistent upregulation/downregulation in both bioinformatics and experimental models. DOCK10 and NUP160, however, exhibit conflicting trends between bioinformatic predictions and murine validation, requiring further investigation in human clinical samples and mechanistic studies to resolve this discrepancy before their biomarker potential can be confirmed.

## Conflicts of Interest

The authors declare no conflicts of interest.

## Author Contributions

Xueyi Sun and Shaolei Geng are the co‐first authors.

## Funding

The study was supported by the National Natural Science Foundation of China, 82070210; the Major Medical Scientific and Technological Project of Henan Province, SBGJ202001008; and the Youth Talent Cultivation Project of Henan Province, 2024HYTP009.

## Data Availability

The data that support the findings of this study are available on request from the corresponding author. The data are not publicly available due to privacy or ethical restrictions.
